# Analysis of Group Randomized Trials with Multiple Binary Endpoints and Small Number of Groups

**DOI:** 10.1371/journal.pone.0007265

**Published:** 2009-10-21

**Authors:** Ji-Hyun Lee, Michael J. Schell, Richard Roetzheim

**Affiliations:** 1 Biostatistics Core, H. Lee Moffitt Cancer Center & Research Institute, Tampa, Florida, United States of America; 2 Department of Family Medicine, University of South Florida, Tampa, Florida, United States of America; Canadian Agency for Drugs and Technologies in Health, Canada

## Abstract

The group randomized trial (GRT) is a common study design to assess the effect of an intervention program aimed at health promotion or disease prevention. In GRTs, groups rather than individuals are randomized into intervention or control arms. Then, responses are measured on individuals within those groups. A number of analytical problems beset GRT designs. The major problem emerges from the likely positive intraclass correlation among observations of individuals within a group. This paper provides an overview of the analytical method for GRT data and applies this method to a randomized cancer prevention trial, where multiple binary primary endpoints were obtained. We develop an index of extra variability to investigate group-specific effects on response. The purpose of the index is to understand the influence of individual groups on evaluating the intervention effect, especially, when a GRT study involves a small number of groups. The multiple endpoints from the GRT design are analyzed using a generalized linear mixed model and the stepdown Bonferroni method of Holm.

## Introduction

This paper addresses data analysis issues related to group randomized trials (GRTs) with multiple endpoints and a small number of groups. In GRT studies, groups serve as the primary sampling unit in the selection process; groups are randomized into two or more arms, and then responses are measured on individuals within those groups. Typical examples of groups include clinics, schools, work sites, churches, or communities.

GRT is becoming a standard study design to assess the effect of an intervention program for health promotion or disease prevention, especially when the intervention is delivered more efficiently to groups than directly to individuals [1].

Consider the data from a study of the cancer Screening Office System (cancer SOS) [2], which motivated this research and are analyzed herein. In the study, eight primary care clinics were randomly selected and assigned to either the intervention or control arms. Then, patients from the clinics were randomly selected and a chart review was conducted to assess whether or not they took a given cancer screening test sometime during the preceding year. This assessment was made at three time points: baseline, 12 and 24 months post intervention.

In contrast to standard randomized clinical trials in which individuals are the sampling units for randomization, GRT designs have additional analytical problems. The major problem is due to the expected positive intraclass correlation (ICC) among observations from individuals in the same group. In addition, the number of groups involved in GRT studies is generally quite small, and thus problems arise in obtaining an accurate estimate of the ICC. In spite of its difficulties, however, the GRT approach may sometimes be the only feasible approach; and if the characteristics of the GRT design are not properly accounted for in the analysis, the statistical significance of the intervention effect is typically overestimated, resulting in misleading public health information.

Data analysis issues of GRTs have been intensively discussed by health science researchers [1], [3]–[5] and a number of studies that used inappropriate statistical analysis methods have been identified [4], [6]–[10]. Recently, a statement from the Consolidated Standard of Reporting Trials (CONSORT) group emphasized the major analytical problems associated with GRTs, and recommended guidelines for reporting about them [6]. Many journals now require that the reports conform to the CONSORT guidelines. We believe that further education regarding the proper analysis of GRT-type data is necessary.

Analytically, it is most straightforward for a clinical trial to have a single primary question. For some research, however, multiple primary endpoints are important and relevant scientifically, medically, or for public health purposes, as it is often difficult to fully assess the efficacy of a new intervention using a single endpoint. Moreover, these studies are often expensive in both cost and effort, so researchers would like to get answers to many questions with a given study, if feasible. For example, the cancer SOS intervention study was designed to increase the use of three major cancer screening tests. For each patient, evaluations of the intervention were made for each test. Thus, patients were measured for the three tests as co-primary endpoints to better characterize the efficacy of the intervention, leading to an outcome variable that is a vector of three responses.

Multivariate analysis involving multiple responses per subject is a major research area in statistics, and several statistical computing packages (e.g., SAS, S-PLUS) have software procedures for analyzing such data. In spite of recent progress though, multivariate analysis is still not a standard approach for analysts who do not routinely use mixed models. Although many papers have explained the theoretical basis for multivariate analysis, few practical introductions to GRTs with multiple endpoints have been written, especially in the context of public health medicine.

Generalized linear mixed models (GLMMs) [11], [12] provide a suitable framework for handling a GRT design. In this paper, we show how to use GLMMs to analyze GRT data arising from a randomized cancer prevention trial. We can incorporate covariates into GRT analysis by formulating the problem in the context of GLMMs.

When a study involves multiple endpoints addressing equally important objectives of the proposed intervention, the potential for drawing false positive conclusions exists unless an appropriate adjustment for multiplicity is used to control the overall statistical error rate. Several possible approaches exist, with appropriateness depending upon the study design. In this study, the multiple endpoints will be adjusted using the stepdown Bonferroni method, which we will refer to as Holm's method [13].

Use of a small number of groups in a GRT raises additional concerns regarding the statistical analysis of the intervention effect. Also, statistical power of a GRT generally depends more on the number of groups randomized than on the average number of individuals within a group [3]. Unfortunately, due to logistics and cost, a GRT commonly involves a small number of groups. From an analytical perspective, a GRT involving few groups intensifies the effect that any single group's behavior has on the overall intervention effect. Thus, it is critical for investigators to understand each group's influence, which poses greater problems if one group is markedly different than the others. In this study, we will develop an index of extra variability to investigate group-specific effects on response. The index is designed to gauge the heterogeneity of response among the individual groups and provide insights into their influence on the GRT study.

This article provides practical advice and methodology for analyzing GRTs data, especially with multiple endpoints and a small number of groups. The overarching goal of this work is to disseminate statistical knowledge for public health benefit. This paper can be viewed as a tutorial for researchers who have little theoretical background or practical experience analyzing GRTs data, especially with multiple endpoints and a small number of groups. Mathematical details are avoided unless needed to illustrate important concepts.

## Methods

### Group randomized trials and statistical issues

A GRT is a randomized clinical trial to investigate an intervention. Unlike standard randomized clinical trials, however, the intervention is delivered to individuals through “groups”, which are assigned to either intervention or control arms. Responses are measured on individuals within those groups nested in the arms. Consequently, the responses for individuals within the same group are expected to be positively correlated. This correlation is called the intraclass correlation coefficient (ICC), and is denoted by 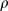.

Although 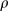 in most GRTs is usually rather small, this dependence can substantially influence the design and analysis of the GRT. If the ICC is ignored, the point estimate of the intervention effect is not affected. However, statistical inferences through the standard errors, p-values, and confidence intervals can be substantially affected.

To explain the concept of the ICC simply, suppose we conduct a nested cross-sectional GRT design. Let the two arms have the same number of groups, 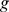, and all groups have the same number of members, 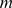, following Murray's notation [1]. In addition, suppose we measure the endpoint at least two times post intervention. The total variance of the response 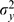 is defined as 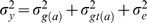, where 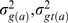, and 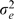 are the between-group, group-by-time interaction, and within-group variances, respectively. Here, 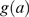 and 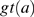 represent group and interaction between group and time are nested within arm. Then, the ICC is defined as
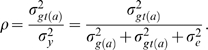
(1)


Note that the ICC varies depending upon the endpoint, the design, and the analyses. For example, with a nested cohort study in which the endpoints are repeatedly observed over time from the same subject, the ICC above will include the within-subject variance over time.

As seen in equation (1), ICC reflects the proportion of the total variance explained by the variance of the group-by-time interaction. The variance of the group mean in a GRT is then,
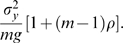



Due to the GRT design, the variance includes a multiplicative factor 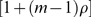 called the variance inflation factor (VIF) [3]. Note that VIF = 1 if 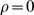. If 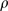 is greater than 0, ignoring the VIF results in underestimating the standard error of the intervention effect, and hence overestimating the statistical significance of the intervention effect.

As equation (1) shows, estimation of the ICC requires estimates of the between-group variance and the group-by-time interaction variance, with the number of groups as degrees-of-freedom. Primarily for logistical reasons, GRTs commonly have few groups (e.g., the four clinics per arm used in the cancer SOS study), resulting in a small number of degrees-of-freedom (df). This small df will be used to estimate the standard error for the intervention effect, and will yield an inflated Type I error. Further, this will reduce the statistical power of the test. Consequently, both the design stage and the analysis stage should account for the GRTs' characteristics.

### Analysis of GRTs using GLMM

In this section, we briefly review the generalized linear mixed model (GLMM) to analyze GRTs. Let's assume that a vector of binary response data 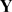 follows a Bernoulli distribution with unknown parameter 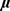. The response probability, 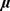, needs a link function so that all possible values of a set of linear predictors map into the interval between 0 and 1. This is accomplished by using the log of the odds of 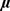, called the logit link function, written by
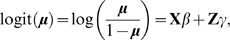
(2)where 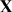 is a matrix of observed explanatory variables and 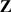 is a matrix for the random effects. The vector 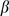 contains the unknown fixed effects that need to be estimated, while the vector 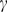 of random effects is not estimable, and is assumed to be normally distributed with mean 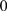 and a variance matrix. The variance of the binary data is defined as,
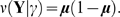
(3)


We analyze the cancer SOS study by testing the intervention effect for the multiple endpoints in a global fashion. Specifically, for each of the three screening tests, let 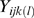 be the binary response (yes/no) and 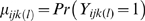 be the probability of taking a screening test for the 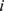
th member nested within the 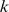 th clinic and the 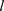 th arm and observed at the 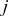 th time. The odds ratio for the effect of intervention can be obtained using a logistic regression model based on equation (2), and it is given by
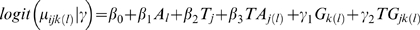
(4)where 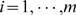 patients; 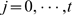 times; 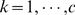 groups; and 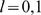 arms. Time is modeled as a continuous variable. In the model, 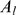 is the intervention indicator (1 for intervention, 0 for the control) that estimates the difference between the intercepts, 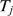 is the 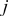 th time point, and the coefficient represents an average slope for the control arm, and 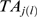 is the time-by-intervention interaction that is the main test of interest as it tests for the average departure from the slope due to the intervention arm.

In order to account for the expected ICC, group-related factors were included in the analysis as random effects: the random effect of the 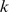 th group nested within arm 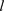, denoted 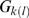, as a group-specific intercept and the random effect of the combination of the 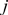 th time and the 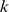 th group nested within arm 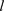, denoted 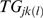, as a group-specific slope. 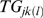 accounts for the possibility that the random effect 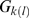 may not have an identical distribution at each time point. These random effects allow for correlation among members within a group and for correlation among members within a group 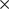 time combination. We assume that the simple diagonal covariance matrix models a different variance component for each random effect, although other covariance matrices are possible. The estimated odds ratios for the fixed effects are then given by 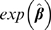. More precisely, the intervention effect is tested by considering the statistical significance of the interaction term, 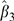. For the final analysis, we added fixed effects for the baseline covariates, age (as a continuous variable) and race (a categorical variable, as shown in Table 1), to the above model to adjust for possible confounding factors.

**Table 1:** Baseline characteristics at individual level by arm (4 groups per arm).

	Control Arm		Intervention Arm		
Characteristics	n = 467	%	n = 468	%	p-value
**Age (years)**					0.270
Under 50	5	1.1	7	1.5	
50–59	235	50.3	258	55.1	
60 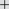	227	48.6	203	43.4	
**Race-ethnicity**					0.014
White	222	47.5	200	44.0	
Black	114	24.4	157	33.6	
Hispanic	120	25.7	98	20.9	
Other	11	3.2	7	1.5	
**Marital status**					0.005
Married	106	22.7	145	31.0	
Non-married	361	77.3	323	69.0	
**Primary language**					0.263
English	361	77.3	376	80.3	
Non-English	106	22.7	92	19.7	
**Health insurance**					0.506
County Health Plan	287	61.5	277	59.2	
Medicaid	65	13.9	79	16.9	
Medicare	88	18.8	80	17.1	
Other	27	5.8	32	6.8	
**Smoking status**					0.706
Smoker	114	24.4	120	25.6	
Non-smoker	353	75.6	348	74.4	
**Health maintenance**					
**visit in past yr**					0.049
Yes	268	57.6	239	51.1	
No	197	42.3	229	48.9	

When a GRT involves a small number of groups, a few statistical methods are available to adjust the degrees-of-freedom in mixed models. For example, Kenward and Roger [15] proposed a general purpose method based on restricted maximum likelihood. Their method uses an adjusted F-statistic that reduces the small sample bias. Its distribution is approximated by the F-distribution with an approximate denominator degrees-of-freedom. This method is easily implemented in some commercial statistical packages including SAS.

Once the model (4) is fitted, the ICC and the VIF can be estimated based on the equation (1), with the estimated variances for the within group, between groups, and a between group-by-time interaction.

Several possible approaches exist to adjust for multiplicity due to multiple endpoints, and the appropriateness depends upon the study design. The adjustment approach could be very conservative (e.g., Bonferroni method) or less conservative (e.g., Hochberg or Hommel method). We prefer Holm's method [13], also known as the stepdown Bonferroni method (a modification from the Bonferroni procedure), as it controls the familywise error rate (FWE) under very general conditions. More powerful methods rely on additional assumptions, and these methods do not always control the FWE. Holm's method is now being widely used and has been cited over 3,000 times to date (www.isiknowledge.com). We refer readers to Brown and Russell [16] for detailed comparisons of various multiple testing procedures. We fit a GLMM for each endpoint and obtain a p-value corresponding to 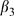 of 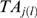 from equation (4). These marginal p-values are then adjusted for multiplicity, using Holm's method (implemented in several statistical packages, including SAS).

### Group and time-specific influences

While VIF provides an interpretation of the overall variance inflation due to a GRT design, it is critical to understand the influence of individual groups on evaluating the intervention effect, especially, when the number of groups is small.

Let us define the change on each endpoint within a particular interval as,
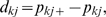
where 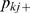 is the estimated probability of a success (e.g., taking a screening test) at time 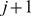, where 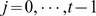 for the 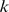
th group with 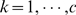. For notational simplicity, we skip the index 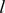 for an arm under this subsection. For a specific time interval, the mean of 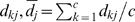, and the standard deviation 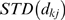, quantifying the amount of variability about 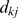, are computed.

If we assume that the responses at two time points are independent, the nominal variance of the average change 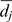 is based on the binomial distribution. We denote the standard deviation of this binomial variance by 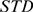. It is obtained as
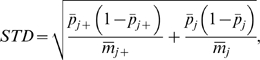
(5)where 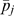 is the estimated sample mean of the proportions computed over all groups in each arm, and 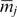 is the average number of individuals within arm at time 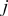. Similarly, 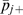 and 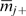 represent the analogous values for time 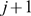. Using 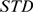, the associated 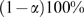 confidence interval for 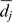 can be computed.

As an index of extra variability (IEV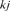), 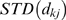 is compared to the variability based on the binomial distribution, using the ratio;
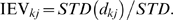



The IEV index describes how spread out the individual group's responses are from the mean, within an arm for a particular period. IEV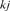 values close to 1 can be interpreted as an indication of strong similarity in response across the groups.

## Results

### Cancer SOS intervention

Roetzheim and others [2] developed a low-cost office-systems intervention called cancer Screening Office Systems (cancer SOS), for primary care clinics serving disadvantaged populations. Disadvantaged populations were defined as patients: 1) belonging to racial or ethnic minorities, 2) of low socioeconomic status, 3) uninsured, or 4) insured by Medicaid. The scientific question for this study was whether or not the intervention prompts patients to take the cancer screening tests described in the next paragraph. In previous reports [2], [14], both men and women were included in the analyses. The present study focuses only on women.

The intervention was implemented in a county-funded health insurance plan in Hillsborough County, FL from 2002 to 2004. Eight clinics were randomly selected, and each was randomly assigned to either the intervention or control arms.

The intervention included a cancer screening checklist completed by patients which indicated whether or not they were due for screening. The intervention targeted three cancer screening tests: Papanicolaou (Pap) smears, mammograms, and fecal occult blood tests (FOBT). It is generally recommended that each of these tests be performed annually for women who are age 50 or older. One hundred fifty patient's charts from the clinics were randomly selected and abstracted to obtain the demographic and clinical variables at baseline, and outcome variables at baseline, 12, and 24 months after the intervention. By chance, a few patients were selected more than once. However, as the study was not intended to follow individuals over time, this design is called a nested cross-sectional study rather than a cohort study.

Table 1 provides descriptive statistics of the cancer SOS study sample at the individual level. The table includes p-values from Fisher's exact tests comparing control and intervention arms. Patients attending intervention clinics were more likely to be African American, married, and have fewer health care visits. However, there was no significant difference in age, language, health insurance, or smoking status at baseline.

### Application

This section illustrates applications of GLMMs to a GRT study where the scientific question is whether or not the cancer SOS intervention convinces patients to take cancer screening tests or not. Men were excluded from the analysis in this paper, as two of the three target screening tests applied to women only. Also, the following exclusion criteria for the women were applied: personal history of breast cancer for the analysis of mammography, personal history of cervical cancer or hysterectomy for the analysis of Pap smear screening, and personal history of colon cancer, or colonoscopy of double-contrast barium enema in the previous 10 years for the analysis of FOBT.

In each of eight clinics, patients' usage of the three screening tests (Pap smear, mammography, and FOBT) was assessed at three time points (baseline, year 1, and year 2). The responses were binary (1 if the patient took the test and 0 otherwise). On average, 150 patients per clinic were measured at each time point, with four clinics assigned to each of two arms (intervention or control).

The distribution of each cancer screening test (numbers and percentages) at each time period is summarized by arm in Table 2. The number of eligible women for the Pap smear test was lower than for the other tests due to a substantial number of hysterectomies. As an initial informal analysis, it is useful to examine the screening rates by time. For all tests, the difference between the two arms was lower at the 24-month compared to the 12-month follow-up. For FOBT, the two arms showed a considerable difference at baseline (24 vs. 36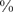 for controls and interventions, respectively), which was maintained over time.

**Table 2:** Distribution of screening (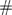 subjects who took screening test/total subjects) by clinic nested within arm and time combination in the cancer SOS study.

	Control Arm				Intervention Arm			
Test	Clinic	Baseline	12 mths	24 mths	Clinic	Baseline	12 mths	24 mths
**Pap Smear**								
	2	31/54	43/68	40/85	5	48/69	50/70	56/79
	7	43/65	23/54	25/66	6	35/51	51/70	43/68
	8	27/65	22/78	28/72	9	37/70	35/79	12/78
	10	47/73	46/77	46/83	11	31/54	40/63	28/69
Total		148/257	134/277	139/306		151/244	176/282	139/294
Rate (%)		57.6	48.4	45.4		61.9	62.4	47.3
Difference								
from Control Arm (%)						4.3	14.0	1.9
**Mammography**								
	2	64/89	87/107	80/116	5	95/114	108/127	82/113
	7	105/124	85/116	79/111	6	88/116	79/115	91/122
	8	67/115	55/111	59/118	9	57/107	82/119	54/119
	10	101/116	95/120	83/117	11	85/118	95/119	92/120
Total		337/444	322/454	301/462		325/455	364/480	319/474
Rate (%)		75.9	70.9	65.1		71.4	75.8	67.3
Difference								
from Control Arm (%)						−4.5	4.9	2.2
**FOBT**								
	2	2/98	11/120	5/118	5	52/118	54/127	44/119
	7	36/126	15/119	19/112	6	6/119	67/119	41/126
	8	42/117	4/116	9/122	9	40/108	4/118	17/122
	10	30/121	29/125	26/117	11	67/118	68/122	37/122
Total		110/462	59/480	59/469		165/463	193/486	139/489
Rate (%)		23.8	12.3	12.6		35.6	39.7	28.4
Difference								
from Control Arm (%)						11.8	27.4	15.8

Time unit is in months (mths).

**Figure 1:**
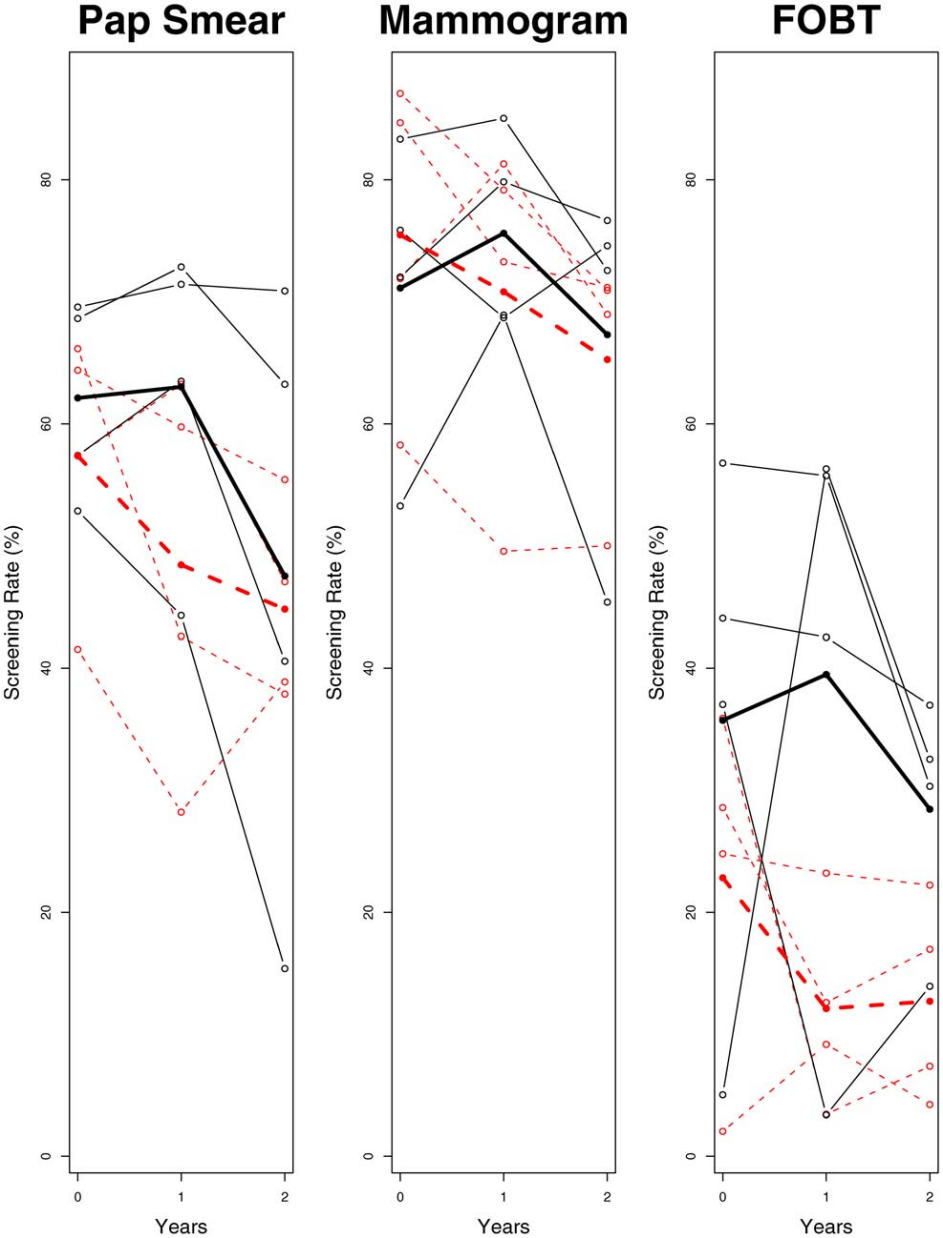
Screening rate in percentage by each clinic (four clinics per arm) for each test across years (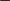: intervention clinics and 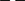: control clinics). The bold lines within each graph represent the average screening rate for each arm respectively.

Figure 1 depicts the data for the three screening tests plotted against time for the 8 clinics. The screening rate trajectories vary among clinics (solid and dot lines represent intervention and control groups, respectively). For the intervention arm, the screening rates increased slightly at year 1 for all three tests, but had declined by year 2. By contrast, the 1-year screening rate declined for all tests in the control arm. Individual clinic trends were fairly similar for mammography and Pap smears, but strikingly different for FOBT. For the most part, the screening rates for interventions were higher than those for controls.

The results of the analysis are shown in Table 3. Age and race (for mammography), and race (for Pap smear) were not significant and were removed from the final model. None of the screening tests showed statistical significance at 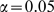 for the intervention effect (Intervention 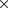 Time). Notably, this result differs from the original analysis [2], [14], which inappropriately ignored the GRT design: those studies reported that the intervention was significant for Pap smear and FOBT screening tests after one year and two years, respectively.

**Table 3:** Summary results of the univariate GLMM analyses for the cancer SOS study.

Test	Estimate	Standard Error			Adjusted
Fixed Effect	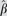	S.E. 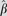	DF	p-value	p-value
**Pap Smear**					
Intercept	2.937	0.569	303.3	0.0	
Intervention	0.385	0.308	6.7	0.25	
Time	−0.022	0.014	6.1	0.17	
Intervention 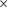 Time	−0.006	0.020	6.4	0.78	1.0
Age	−0.041	0.009	1654	0.0	
Race:Hispanic	0.288	0.114	1654	0.012	
**Mammography**					
Intercept	1.201	0.532	6.9	0.092	
Intervention	−0.144	0.425	6.9	0.71	
Time	−0.279	0.082	6.2	0.015	
Intervention 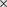 Time	0.180	0.115	6.0	0.17	0.51
**FOBT**					
Intercept	−2.282	0.632	30.5	0.011	
Intervention	0.914	0.583	5.6	0.17	
Time	−0.441	0.299	6.3	0.19	
Intervention 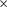 Time	0.221	0.416	5.9	0.61	1.0
Age	−0.041	0.009	1654	0.0	

Table 4 shows the estimated covariances of the random effects from the above model as well as the estimated ICC and VIF values. Even though the ICCs might appear to be modest in size, the VIFs are substantial. This indicates that the variances of the intervention effects range from 1.3 to 19.9 times larger than they would have been with random assignment of individual members, providing a major explanation for the differences in findings from the original analyses.

**Table 4:** Estimates of covariance-parameters and intra-class coefficient (ICC) & variance inflation factor (VIF) from the univariate GLMMs.

Test	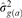	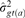	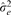	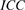	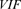
Pap Smear	0.139	0.085	1.00	0.069	5.7
Mammography	0.321	0.004	1.00	0.003	1.3
FOBT	0.632	0.312	1.01	0.160	19.9

**Figure 2:**
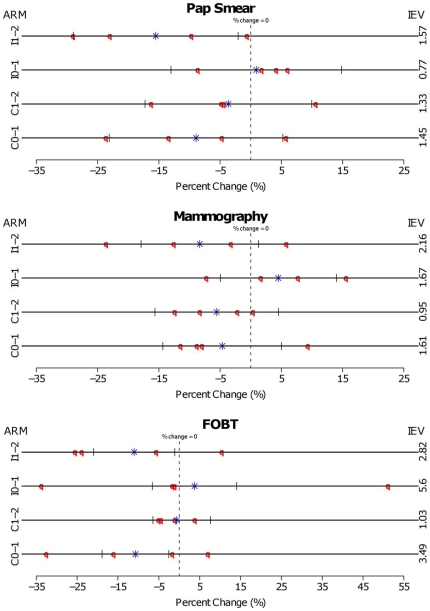
Percent change (%) between two time points by each clinic and arm for each screening test, with index of extra variability (IEV): Arm I0-1,I1-2, C0-1, C1-2: Intervention (I) and Control (C) between baseline and year 1 (0–1) and between year 1 and 2 (1–2). 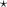
: Mean of difference between two time points across clinics. : Individual clinics' difference in screening proportion between two time points. 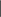 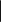 : 90 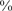 CI based on binomial distribution.

The huge differences of VIF across screening tests are more clearly explained in Figure 2, which depicts the change of screening rates (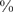) between two time points by clinic and arm for each screening test. The symbol of star and the brackets represent the mean change across clinics and the 90% confidence intervals (CIs) based on equation (5). Overall, for the Pap smear test, the changes for the individual clinics (denoted by circle symbols) were near the mean, and the index of extra variability (IEV) relatively close to 1. By contrast, the IEV values were much higher for the FOBT test, except for the 1–2 year change for the control arm. Specifically, for the intervention arm with the period of 0 to 1 year (IEV = 5.6), two of the four clinics showed marked distances from the group mean at −34% and 51%. The IEV values for mammography were usually somewhat higher than they were for the Pap smear test, in spite of the fact that the VIF for the mammography test was much lower (1.3. vs. 5.7). This contrast can be explained by the relative lack of consistency in when the shifts occurred for Pap smears; the rate was fairly stable from baseline to one year, but decreased about 15% in the second year for the intervention groups, whereas the most of the decline among the control groups was in the first year. The FOBT, likewise, shows large shifts whose pattern differs by treatment arm.

## Discussion

GRT designs are becoming increasingly important in public health interventions, as it is often easier to randomize at the group level. Investigators are often surprised by the degree to which information is attenuated due to intraclass correlation, even though ICC levels are often quite low in practice. Thus, GRT designs are usually improved by having as many groups as are logistically possible. We have developed an index of extra variability (IEV) with a corresponding graphical presentation for understanding in greater detail how the effects from individual groups influence the overall findings. We believe that the IEV concept will assist researchers in the planning future GRT studies.

The results of the cancer SOS GRT study were disappointing. Further, the primary investigator of the study, a non-statistician, was understandably confused at the discrepancy in findings based on the statistical method employed. The original analysis, a generalized estimating equations (GEE) [17] approach for each individual endpoint [2], [14], found that the intervention increased all three screening tests at one year follow-up, and had a persistent effect on the mammography at two year follow-up.

While the GEE method allows for correlation among patients within a group, it is inappropriate for GRT study designs when the number of clinics is small, with less than 20 groups per arm, as noted by Murray et al. [5] and Bellamy et al. [18]. It is known that the GEE approach generally yields biased estimates of the variance of fixed effects when the number of groups/clusters is small [19], [20]. Recently, standard software packages are beginning to correct for the bias issues for GEE with small samples. In addition, while commonly ignored, the univariate approach for each individual endpoint requires an adjustment for multiplicity.

While analyzing the cancer SOS study, we faced a few additional analytical issues that should be discussed. As the endpoints were measured at three times, some patients were seen at multiple timepoints by chance. Among the patients surveyed at baseline, 22% were seen at year 1, and 8% were selected at all three timepoints. Ideally, the expected intra-patient correlation, which exists as a consequence of repeated measurements across time, should be adjusted for when performing statistical inference. However, in this study we did not consider this additional source of correlation. The effect of the possible correlation across time is unknown and needs further investigation. Also, each patient had three endpoints, resulting in another source of intra-patient correlation. As the univariate approach ignores the stochastic dependence among the individual endpoints, it may yield conservative results. To account for this type of intra-correlation, one may use a multivariate approach; all three endpoints per subject unit are simultaneously analyzed by adding a random effect for individuals. By accounting for the correlation among endpoints, improved power to detect the overall intervention effect is expected. In addition, this multivariate approach needs no multiplicity adjustment, since only one test is being carried out. However, it should be noted that if the endpoints describe unrelated aspects of the individual response, or if there is considerable discrepancy across endpoints, this multivariate approach is not a reasonable analytical strategy. In this case, individual tests that are suitably adjusted, as we have done here, should be used. The cancer SOS showed striking differences in VIF across the three endpoints (i.e., 1.3 to 19.9); thus, averaging the three endpoints provides no reasonable interpretation.

What appears to have happened with the SOS GRT is that the intervention groups were either stable or had increases in the first year, while the control groups declined. However, these modest gains were short-lived, and resulted in large year 2 drops for the intervention groups. The GRT was not designed to assess this kind of change, or cope with this degree of heterogeneity, given the small number of groups. As VIF, IEV, and the graphical presentation in Figure 2 all provide different, albeit related, information for GRTs, we believe that they all have a useful role in their analysis. Selecting an appropriate number of groups and the number of subjects sampled per group in GRTs depends on several factors: the endpoint variable type, the method of the analysis, the expected effect size of intervention, and the estimated intra-cluster correlation coefficient. The sample size issue of GRTs is another topic that requires separate discussion; we refer to Donner and Klar [3], and Murray [1] for details.

Our hope is that this paper will contribute to the responsible analysis of GRTs, thereby helping with the scientific accuracy of research findings. Multivariate analysis for multiple endpoints in GRTs is obviously one area that we need to continue further investigation. Further research on small group issues also is warranted at both the design and analysis stages.

The SAS code used for this article is available at the first author's web: http://personal.health.usf.edu/jlee2/software. Note that it also includes a Newton-Raphson optimization option to deal with convergence problems, which we otherwise frequently ran into for moderately complex mixed models.
